# Marginal Zone B Cells Assist With Neutrophil Accumulation to Fight Against Systemic *Staphylococcus aureus* Infection

**DOI:** 10.3389/fimmu.2021.636818

**Published:** 2021-05-10

**Authors:** Li-Wen Lo, Chia-Wei Chang, Ming-Feng Chiang, I-Ying Lin, Kuo-I Lin

**Affiliations:** ^1^ Graduate Institute of Immunology, College of Medicine, National Taiwan University, Taipei, Taiwan; ^2^ Genomics Research Center, Academia Sinica, Taipei, Taiwan

**Keywords:** marginal zone B cell, IL-6, neutrophil, *Staphylococcus aureus*, FRET

## Abstract

In addition to regulating immune responses by producing antibodies that confer humoral immunity, B cells can also affect these responses by producing cytokines. How B cells participate in the clearance of pathogenic infections *via* functions other than the production of pathogen-specific antibodies is still largely unknown. Marginal zone (MZ) B cells can quickly respond to bacterial invasion by providing the initial round of antibodies. After a bloodborne bacterial infection, neutrophils promptly migrate to the MZ. However, the mechanisms regulating neutrophil accumulation in the MZ during the initial phase of infection also remain obscure. Here, we found that MZ B cell-deficient mice are more susceptible to systemic *Staphylococcus aureus* (*S. aureus*) infection compared with wildtype mice. The expression levels of interleukin (IL)-6 and CXCL1/CXCL2 in MZ B cells increased significantly in mice at 3–4 h after infection with *S. aureus*, then decreased at 24 h post-infection. After systemic *S. aureus* infection, splenic neutrophils express increased CXCR2 levels. Our results from confocal microscopy imaging of thick-section staining demonstrate that neutrophils in wildtype mice form cell clusters and are in close contact with MZ B cells at 3 h post-infection. This neutrophil cluster formation shortly after infection was diminished in both MZ B cell-deficient mice and IL-6-deficient mice. Blocking the action of CXCL1/CXCL2 by injecting anti-CXCL1 and anti-CXCL2 antibodies 1 h before *S. aureus* infection significantly suppressed the recruitment of neutrophils to the MZ at 3 h post-infection. Compared with peptidoglycan stimulation alone, peptidoglycan stimulation with neutrophil co-culture further enhanced MZ B-cell activation and differentiation. Using a Förster resonance energy transfer by fluorescence lifetime imaging (FLIM-FRET) analysis, we observed evidence of a direct interaction between neutrophils and MZ B cells after peptidoglycan stimulation. Furthermore, neutrophil depletion in mice resulted in a reduced production of *S. aureus*-specific immunoglobulin (Ig)M at 24 h post-infection. Together, our results demonstrate that MZ B cells regulate the rapid neutrophil swarming into the spleen during the early phase of systemic *S. aureus* infection. Interaction with neutrophils assists MZ B cells with their differentiation into IgM-secreting cells and contributes to the clearance of systemic bacterial infections.

## Introduction

There is a growing body of evidence demonstrating that not only are B cells capable of producing antibodies but these cells also have a regulatory role in immune responses *via* cytokine production (1). B cells develop in the bone marrow, after which immature B cells travel to the secondary lymphoid tissues to further mature into transitional B cells (2). Transcription factor recombination signal binding protein-J (RBP-J) regulates downstream gene expression activated by Notch receptors. Notch–RBP-J signaling determines the development of mature B cells in the spleen into follicular B cells or marginal zone (MZ) B cells (3). MZ B cells are located outside the marginal sinus at the interface between the white pulp and red pulp in the spleen. Compared with follicular B cells, MZ B cells are in a preactivated state and thus respond faster to foreign pathogens (4–7). They provide timely defense in the initial phase of bloodborne microbial infection by detecting pathogens through toll-like receptors and B-cell receptors (8). MZ B cells integrate the roles of sensing pathogens and effector cells during T cell-independent immune responses, then promptly differentiate into plasma cells that secrete protective antibodies. MZ B cells can also participate in T cell-dependent immune responses by capturing bloodborne pathogens and transferring them to the follicular area of the spleen. These cells can rapidly produce IgM antibodies following appropriate stimulation and subsequently differentiate into short-lived plasma cells that aid in early immune responses, thus filling the protective gap between the innate and adaptive immune responses (9–11).

Interleukin (IL)-6 is the main inflammatory cytokine released by B cells during the initial stage of infection (12, 13). Studies have shown that IL-6 has both proinflammatory and anti-inflammatory properties. IL-6 binds to the membrane-bound receptor IL-6R, then forms a signaling complex *via* a glycoprotein 130 (gp130) homodimer to stimulate intracellular signaling pathways (14–16). This critical regulator of innate immunity promotes the transition of leukocyte recruitment during acute inflammation from neutrophils to monocytes *via* inducing the expression of chemokines, such as MCP-1 and CXCL8, in endothelial cells (17). IL-6 has also been shown to play an anti-inflammatory role through suppressing neutrophil recruitment during acute inflammatory responses (18). Whether IL-6 released by B cells participates in the regulation of neutrophil recruitment into the MZ during the early phase of bloodborne pathogen infection remains to be determined.

Neutrophils are generally short-lived, with a circulation half-life of 6–8 h (19). They form the main innate immune cell population that can quickly eliminate pathogenic invasion. Once neutrophils are recruited to the site of infection through the leukocyte adhesion cascade, these cells may function for 1–2 days in the tissues before undergoing apoptosis (20, 21). However, there are many factors capable of prolonging neutrophil survival during inflammatory responses, such as granulocyte-macrophage colony-stimulating factor, granulocyte colony-stimulating factor (G-CSF), tumor necrosis factor-α (TNFα), interferon γ, IL-6, and bacterial or fungal products (22, 23). Neutrophils show different phenotypes and functional responses after being primed by microbes (24). Several studies have indicated that certain neutrophils colonize in the red pulp region of the spleen in a noninflammatory state. When infection occurs, they quickly migrate to the infection site, together with circulating neutrophils, and release active molecules to control microbial infection. These splenic neutrophils provide critical help by releasing cytokines to promote antibody production by MZ B cells and immunoglobulin class switching (25–27). The infiltration of neutrophils into inflamed and infected tissues is initiated by a small number of neutrophils. They respond to the initial danger signals by secreting proinflammatory mediators and forming small clusters. Within a few minutes, the accumulation of a large number of neutrophils and the formation of neutrophil clusters then promote their coordinated effector functions in subsequent innate immune responses (28).

Neutrophils can interact with other cell types, including dendritic cells, macrophages, B cells, and T cells, *via* various receptors, such as chemoattractant receptors, fragment crystallizable (Fc)-receptors, cytokine receptors, Toll-like receptors, and C-type lectin receptors (29–31). Despite their short lifespan, neutrophils play a vital role in host defense against bacterial infection and regulate adaptive immunity (32). However, the details of the interaction between MZ B cells and neutrophils during the response to bloodborne microorganism infection remain largely unknown. *Staphylococcus aureus* is a Gram-positive human pathogen that is a cause of serious infections within communities and hospitals. Systemic *S. aureus* infections can cause severe disease manifestations, such as sepsis (33). Here, we investigate the regulatory loop between MZ B cells and neutrophils during acute systemic infection with *S. aureus*.

## Materials and Methods

### Mice

All mice used in this study had a C57BL/6 genetic background. Wildtype (WT) C57BL/6 mice were purchased from BioLASCO and National Laboratory Animal Center in Taiwan (Taipei, Taiwan). The RBP-J^f/f^ × CD19-Cre^+^ conditional knockout (RBP-J CKO) mice were kindly provided by Dr. Tasuku Honjo (Institute for Advanced Study, Kyoto University, Japan). The LysM-eGFP mice expressing enhanced green fluorescent protein (eGFP) in granulocytes were provided by Dr. Ellen Robey (UC Berkeley, US) (34). The IL-6 knockout (IL-6 KO) mice were purchased from The Jackson Laboratory. All mice were bred and kept under specific pathogen-free conditions in Academia Sinica Animal Care Facility before infection with *S. aureus* in P2/P3 animal facility. Animal experimental procedures and the use of the animals were approved by the Institutional Animal Care and Use Committee (IACUC) of Academia Sinica.

### Flow Cytometry Analysis

Single-cell suspensions of splenocytes were stained with the following antibodies: anti-CD138-Brilliant Violet 421 monoclonal antibody (mAb; clone 281-2, Biolegend), anti-Ly6C-FITC mAb (clone HKL4, Biolegend), anti-CD21/35-APC mAb (clone 7E9, Biolegend), anti-CD23-PEcy7 mAb (clone B3B4, Biolegend), anti-B220-APCcy7 mAb (clone RA3-6B2, Biolegend), anti-Ly6G-FITC mAb (clone 1A8, Biolegend), anti-CD11b-PE mAb (clone M1/70, Biolegend), anti-CD69 mAb (clone H1.2F3, BD Biosciences), and anti-CD86 mAb (clone GL1, BD Biosciences). The applied staining methods adhered to the recommendations in “Guidelines for the use of flow cytometry and cell sorting in immunological studies” (35). Cells were fixed and permeabilized using Foxp3/Transcription Factor Fixation/Permeabilization Kit (Invitrogen; ThermoFisher REF 00-5523-00), and then stained with anti-IL-6 mAb (clone MP5-20F3, Biolegend) with anti-IL-6 APC mAb (clone MP5-20F3, Biolegend), anti-CD21/35-FITC mAb (clone 7E9, Biolegend), and anti-CD23-Brilliant Violet 421 mAb (clone B3B4, Biolegend) for detecting intracellular IL-6. Neutrophils (Ly6G^hi^CD11b^hi^), MZ B cells (B220^+^CD21^hi^CD23^lo^), and plasma cells (CD138^+^ or CD138^+^Ly6C^+^) were analyzed by using a BD FACSCanto II flow cytometer. In the co-culture experiments, mouse spleen MZ B cells (B220^+^CD21^hi^CD23^lo^) were sorted by a BD FACSAria sorter. Flow cytometric data were analyzed by BD FlowJo software.

### *S. aureus* Infection and Preparation of *S. aureus* Lysates

Eight- to ten-week-old mice were intravenously injected with 2.5–5 × 10^6^ colony-forming units (CFU) of *S. aureus* (regular strain ATCC25923), after which the mouse survival and bodyweight changes were monitored daily for up to 10 days. In some experiments, mice were intraperitoneally administered 400 µg of anti-Ly6G antibody (clone 1A8, BioXCell) to deplete neutrophils or 400 µg of isotype control antibody (clone 2A3, BioXCell) as a control at 24 h before *S. aureus* infection. Four hours later, MZ B cells were isolated by using a FACSAria flow cytometer (BD) to perform the subsequent analysis. *S. aureus* lysates were prepared in the B-PER Reagent (Thermo Fisher Scientific) in accordance with the manufacturer’s protocol. Briefly, *S. aureus* was grown overnight in trypticase soy broth, killed by treatment with 3% formalin for 30 min, washed twice with phosphate-buffered saline (PBS), and stored at −80°C before processing. Cell pellets were suspended in 4 ml of B-PER Bacterial Protein Extraction Reagent per gram of wet mass. DNase I (300 µg/ml, BD) was added into a solution containing an EDTA-free protease inhibitor cocktail (Roche) and combined with the bacterial mixture, which was then incubated for 15 min at room temperature on a shaking platform. Bacterial mixtures were subsequently sonicated on ice 10 times for 40 s each, with a 40-s interval between each sonication, by using a Q700 sonicator (QSonica). The resulting crude extracts were centrifuged at 10,000 ×*g* for 20 min at 4 °C, followed by the removal of cell debris.

### Measurement of *S. aureus*-Specific IgM by Enzyme-Linked Immunosorbent Assay (ELISA)

Mice were intravenously injected with *S. aureus* (2.5 × 10^6^ CFU) or left untreated, then sacrificed at the indicated times. The splenic tissue fluids in 1 ml of RPMI medium were harvested by collecting the supernatants resulting from centrifugation, and they were stored at −80°C before processing. A 96-well ELISA plate was precoated overnight with *S. aureus* lysate (70 µg/well), and the splenic tissue fluids were then subjected to ELISA analysis, performed in accordance with the manufacturer’s instructions, to determine the post-infection levels of *S. aureus*-specific IgM in the spleen. The absorbance was detected at 450 nm by a microplate reader (SpectraMax M2).

### Neutrophil Isolation

Splenocytes harvested from mice infected with *S. aureus* (2.5 × 10^6^ CFU) were overlaid on four-layer Percoll gradients (45%-55%-62%-81%) (GE Healthcare). Neutrophils were collected, as previously described, from the interface of the 62%-81% layers after centrifugation at 3,000 ×*g* for 30 min without the use of a brake and were washed with Hank’s Balanced Salt Solution (HBSS) without Ca^2+^ and Mg^2+^ (36, 37). A highly pure neutrophil population (90%–95% Ly6G^hi^CD11b^hi^), as assessed by flow cytometry, was isolated by using negative selection beads (Stemcell Technology) in accordance with the manufacturer’s instructions.

### Microarray Analysis

FACS-sorted MZ B cells (B220^+^CD21^hi^CD23^lo^) and neutrophils isolated by negative selection beads (Stemcell Technology) were purified from mice before and 4 h after *S. aureus* (2.5 × 10^6^ CFU) infection. RNA was extracted by using the RNeasy Mini Kit (Qiagen, Valencia, CA, USA) in accordance with the manufacturer’s instructions. The isolated RNA (100 ng) combined from MZ B cells or neutrophils from multiple mice was subjected to a cDNA microarray analysis using a Clarion™ D Array, mouse (Affymetrix) in accordance with the manufacturer’s instructions. The array data were acquired using a GeneChip Scanner 3000 (Affymetrix) and analyzed by GeneSpring. The raw data were deposited in the Gene Expression Omnibus (GEO) database under the accession number GSE157176. Differentially expressed genes in MZ B cells or neutrophils, with an upregulation or downregulation of ≥1.5-fold from 0 h to 4 h post-infection are shown.

### Real-Time PCR Analysis

An RNeasy Plus Mini Kit (Qiagen) was used to extract the total RNA from sorted MZ B cells or neutrophils isolated by negative selection beads (Stemcell Technology) taken from mice at 0, 4, or 24 h post-infection with *S. aureus*, and cDNA was generated from the resulting RNA by using a High-Capacity cDNA Reverse Transcription Kit (Applied Biosystems) in accordance with the manufacturer’s instructions. The resulting cDNA (10 ng) and gene-specific primer sets were used with the QuantStudio 5 Real-Time PCR System (Applied Biosystems) to perform a qPCR analysis. The primer pairs for SYBR green detection are listed below:

*Cxcl1*: 5’-GCAGACCATGGCTGGGATT-3’ and 5’-TGTCAGAAGCCAGCGTTCAC-3’,
*Cxcl2*: 5’-ACTGCGCCCAGACAGAAGTC-3’ and 5’-CAGTTAGCCTTGCCTTTGTTCAG-3’,
*Cxcr1*: 5’-CCATTCCGTTCTGGTACAGTCTG-3’ and 5’-GTAGCAGACCAGCATAGTGAGC-3’,
*Cxcr2*: 5’-CACTATTCTGCCAGATGCTGTCC-3’ and 5’-ACAAGGCTCAGCAGAGTCACCA-3’,
*Il-6*: 5’-TTCCATCCAGTTGCCTTCTTGG-3’ and 5’-TTCTCATTTCCACGATTTCCCAG-3’,
*Il-10*: 5’-GCTCTTACTGACTGGCATGAGGAT-3’ and 5’-GCTGGTCCTTTGTTTGAAAGAAAG-3’, *Tnf*: 5’-GACCCTCACACTCAGATCATCTTCT-3’ and 5’-CCTCCACTTGGTGGTTTGCT-3’

Relative levels of mRNA were normalized to Actin expression in each sample. *Actin* are 5’-CATTGCTGACAGGATGCAGAAGG-3’ and 5’-TGCTGGAAGGTGGACAGTGAGG-3’.

### Cytokine Measurement

Uninfected mice or mice infected with *S. aureus* (2.5 × 10^6^ CFU) were sacrificed at the indicated times. The splenic tissue fluids were then collected and used in an ELISA analysis to detect the levels of mouse IL-6 (Thermo Fisher Scientific). The absorbance at 450 nm was detected by using a microplate reader (SpectraMax M2). In the *in vitro* experiments, MZ B cells (1 × 10^6^ cells) were sorted by the BD FACSAria system, seeded in 96-well plates precoated with 3% agarose gel in culture medium, then stimulated with 2 µg/ml peptidoglycan (PGN; InvivoGen) for 24 h. In co-culture experiments, neutrophils (1 × 10^6^ cells/well) were seeded in 96-well plates precoated with 3% agarose, after which the sorted MZ B cells (1 × 10^6^ cells/well) were added, and the resulting cultures were incubated for 24 h with or without PGN (2 µg/ml) or heat-killed *S. aureus* (MOI=10) stimulation. The preparation of heat-killed *S. aureus* was performed by incubating bacteria at 80°C for 30 min. The levels of proinflammatory cytokines in the culture supernatants were measured by using Cytometric Bead Array (CBA) Kits (BD Biosciences).

### Confocal Microscopy Analysis of Thick Tissue Sections

In immunostaining experiments, mice were intravenously injected with *S. aureus* (2.5 × 10^6^ CFU) and then sacrificed at the indicated times. In some experiments, mice were intravenously injected with 50 μg each of anti-CXCL1- and CXCL2-neutralizing antibodies (MAB453 and MAB452, R&D Systems) or equal amounts of IgG isotype control antibody (R&D Systems) 1 h before *S. aureus* infection. The spleens were harvested, fixed overnight in freshly prepared 4% paraformaldehyde in PBS, and washed three times for 10 min/wash with PBS. The spleens were then embedded in 4% agarose before being cut into 200-µm sections by using a vibrating microtome (Vibratome) (38, 39). The thick spleen sections were treated with freshly prepared sodium borohydride in PBS (1 mg/ml) three times for 10 min/wash to help reduce the background staining (40), and finally washed with PBS. The sections were then transferred into a permeabilization solution containing 1% Triton X-100 and 2% Tween 20 and incubated overnight at 4°C. Nonspecific binding was reduced by incubating the sections overnight in a blocking solution consisting of 10% normal goat serum in PBS at 4°C. The sections were washed three times with PBS for 10 min/wash at room temperature, then incubated for 2 days at 4°C with the following antibodies: anti-CD45R B220 (1:50 dilution, clone RA3-6B2, Violet Fluor 450, Merck), anti-mouse CD1d-PE (1:50 dilution, clone 1B1, Biolegend), and anti-mouse Ly6G-Alexa 647 (1:50 dilution, clone 1A8, Biolegend) antibodies. The sections were finally washed overnight with PBS in a rotating platform at 4°C, then mounted in Fluormount G (Southern Biotechnology Associates). The images were acquired by the Leica SP8 confocal microscope. Three spleens per experimental group at each timepoint were analyzed.

### Förster Resonance Energy Transfer by Fluorescence Lifetime Imaging (FLIM-FRET) Analysis

The splenic neutrophils isolated from LysM-eGFP mice using negative selection beads (Stemcell Technology) were seeded in precoated dishes containing 3% agarose for 1 h, then co-cultured for 3 h at a 1:1 ratio with MZ B cells that had been sorted using an MZ and FO B Cell Isolation Kit (Miltenyi Biotec) under stimulation with PGN (2 µg/ml). The cells were then harvested and fixed in 4% paraformaldehyde, washed three times with PBS for 10 min/wash, blocked with 1% normal goat serum, and labeled with anti-CD19 primary antibody (1:100 dilution, Abcam) followed by Alexa Fluor 546-labeled secondary antibody (1:100 dilution, Biolegend) to detect MZ B cells. The FRET pairs are eGFP (serving as the donor fluorophore) and Alexa Fluor 546 (serving as the acceptor fluorophore). The Leica SP8 FALCON system was used to perform FLIM-FRET detection in the 488 nm argon laser channel for measuring the change in the fluorescence lifetime of the donor molecule.

### Statistical Analysis

The statistical analyses in this study were primarily conducted by using GraphPad Prism 8 software (GraphPad Software, San Diego USA); *p*-values of <0.05 were considered statistically significant. Presented data are shown as the mean ± standard error of the mean (SEM). Unpaired Student’s *t*-tests were used for comparing the differences between two groups. Comparisons between multiple groups were performed using a one-way ANOVA, followed by Dunnetts’ honestly significant difference *post hoc* test. The differences in mouse survival curves between the two groups were analyzed by a log-rank test.

## Results

### Mice Lacking MZ B Cells Are More Susceptible to *S. aureus* Infection

**Figure 1:**
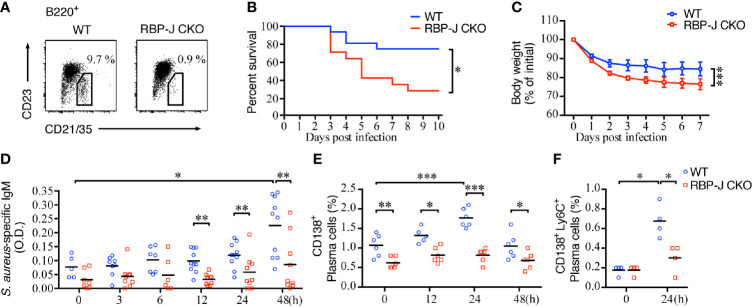
Mortality and *S. aureus*-specific IgM production in mice lacking MZ B cells. **(A)** Splenocytes of RBP-J^f/f^ × CD19Cre^+^ (RBP-J CKO) or WT mice were stained with antibodies against B220, CD21/35, and CD23. MZ B cells were defined as B220^+^CD21/35^hi^CD23^lo^ by flow cytometry. The numbers denote the percentage of MZ B cells in the B220 gate. **(B)** The survival rates of RBP-J CKO and WT mice were recorded daily after the mice were infected with 5 × 10^6^ CFU of *S. aureus*. The survival rate differences were analyzed by a log-rank (Mantel-Cox) test. **(C)** The bodyweight loss of mice after they were infected with 2.5 × 10^6^ CFU of *S. aureus.* The statistical analysis was conducted by performing an unpaired *t*-test (n = 7). **(D)** ELISA analysis using splenic tissue homogenates to measure the levels of *S. aureus*-specific IgM in RBP-J CKO and WT mice at various days after infection with 2.5 × 10^6^ CFU of *S. aureus*. The statistical analysis was conducted by performing an unpaired *t*-test (n = 5–10). **(E)** Differences in the percentages of CD138^+^ plasma cells in RBP-J CKO and WT mice at the indicated timepoints after *S*. *aureus* infection were compared. **(F)** Differences in the percentage of CD138^+^Ly6C^+^ plasma cells in RBP-J CKO and WT mice at 24 h after *S. aureus* infection. The statistical analysis was conducted by performing an unpaired t-test (n = 4). Data are presented as the mean ± SEM. *p < 0.05, **p < 0.01, and ***p < 0.001.

Previous reports indicate that Notch–RBP-J signaling regulates the lineage commitment of MZ B cells but does not affect B1 cells or other lineage cells. The absence of RBP-J leads to the loss of MZ B cells but does not cause defects in B-cell homeostasis, differentiation, or activation (3). Here, we used RBP-J CKO mice that lacked MZ B cells (**Figure 1A**) to study the regulatory role of MZ B cells during infection with the common pathogenic bacteria *S. aureus*. We found that the survival rate after systemic infection with *S. aureus* of RBP-J CKO mice was significantly lower than that of littermate WT mice (**Figure 1B**). Similarly, the bodyweight loss after systemic *S. aureus* infection was more severe in RBP-J CKO mice as compared with that in WT mice (**Figure 1C**). These results indicate that mice lacking MZ B cells are more susceptible to systemic bacterial infections. We noticed that the differences in bodyweight loss between WT and RBP-J CKO mice occurred within 1–2 days after infection, so we wondered whether certain innate immune responses may work together with MZ B cells to clear the bloodborne bacterial infection.

We also checked the amounts of *S. aureus*-specific antibody after systemic infection in WT and RBP-J CKO mice. IgM is known to provide the first line of humoral immunity defense against pathogens and plays an important role during microbial infection (41). Indeed, we found that *S. aureus*-specific IgM production in the spleen increased significantly at 48 h after systemic infection (**Figure 1D**), which is linked with the increases in the percentage of plasma cells in the spleen at 24 h post-infection in WT mice (**Figure 1E**). In contrast, the amounts of *S. aureus*-specific IgM and the frequency of plasma cells produced by RBP-J CKO mice at 12, 24, and 48 h after systemic *S. aureus* infection were significantly lower (**Figures 1D, E**). Although the frequency of plasma cells decreased at 48 h post-infection in WT mice, these mice still had significantly higher percentages of splenic plasma cells compared with RBP-J CKO mice (**Figure 1E** and **Supplementary Figure 1A**). We also compared the frequency of plasma cells by using another more specific marker, CD138^+^Ly6C^+^ (42), at 24 h after *S. aureus* infection. Both approaches showed that the frequency of plasma cells in WT mice was significantly higher than that in RBP-J CKO mice at 24 h after infection (**Figure 1F** and **Supplementary Figure 1B**). These results suggest that MZ B cells contribute to the production of *S. aureus*-specific IgM in the early phase of infection and that the reduced levels of *S. aureus*-specific antibody are associated with the reduced survival of RBP-J CKO mice.

### Cytokine IL-6 and Chemokine CXCL2 Were Upregulated in MZ B Cells After *S. aureus* Infection

**Figure 2:**
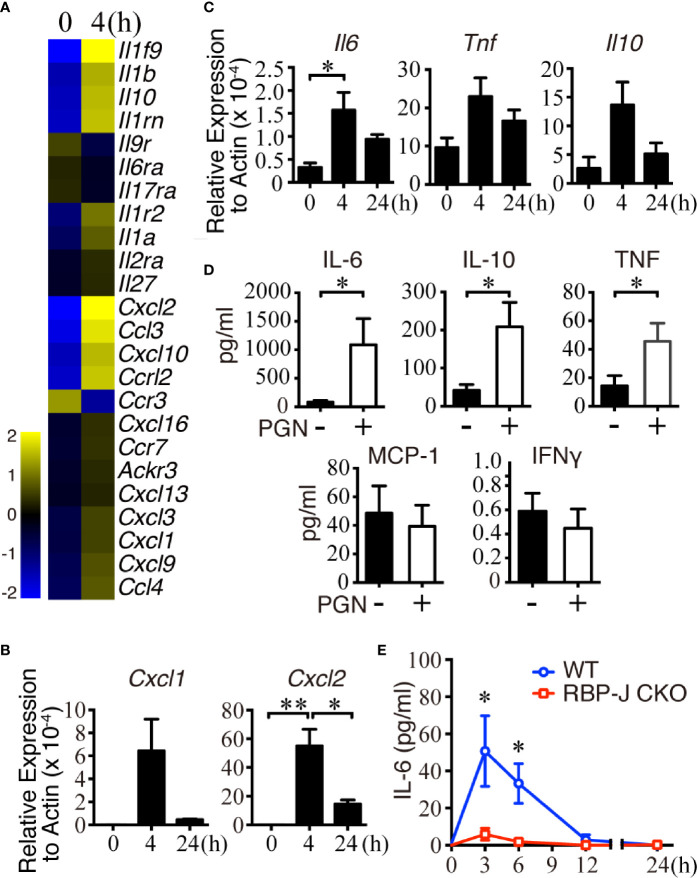
Changes of cytokine and chemokine expression in MZ B cells in the early stage of *S. aureus* infection. **(A)** Changes in the expression of chemokine- and cytokine-related genes in MZ B cells isolated from mice after infection with *S. aureus* (2.5 × 10^6^ CFU) by iv injection, as revealed by a cDNA microarray analysis. Heatmap showing the expression of several chemokine- and cytokine-related genes in MZ B cells. **(B)** RT-qPCR analysis of the *Cxcl1/Cxcl2* mRNA levels in MZ B cells isolated 0, 4, or 24 h after *S. aureus* infection. Results were normalized to the actin mRNA levels. **(C)** RT-qPCR analysis of the *Il6, Il10*, and *Tnf* mRNA levels in MZ B cells isolated 0, 4, or 24 h after *S. aureus* infection. Results were normalized to the actin mRNA levels. **(D)** Cytokines produced by purified MZ B cells treated with or without PGN (2 µg/ml) for 24 h were determined by a Cytometric Bead Array. **(E)** ELISA showing the levels of IL-6 in the splenic tissue homogenates of RBP-J CKO and WT mice at 0, 3, 6, 12, and 24 h after *S. aureus* infection (2.5 × 10^6^ CFU). Statistical analysis was conducted by performing an unpaired *t*-test **(C, E)** and one-way ANOVA **(B, D)**. Data are presented as the mean ± SEM (n = 3 in B, n = 6–7 in C, n = 3–6 in D, and n = 6 in E). *p < 0.05 and **p < 0.01.

B cells can produce a variety of cytokines/chemokines, such as IL-2, IL-4, TNFα, IL-6, IL-10, CXCL1, CXCL10, and CXCL13, in response to stimuli (43, 44). We hypothesized that MZ B cells may produce some cytokines/chemokines to regulate the recruitment of innate immune cells in systemic pathogen clearance, and thus sought to identify changes in the expression of cytokines/chemokines in MZ B cells after systemic *S. aureus* infection. Towards this end, sorted MZ B cells from uninfected mice or mice infected 4 h previously with *S. aureus* were subjected to cDNA microarray analyses. Eleven cytokine genes and thirteen chemokine genes in MZ B cells were found to be differentially upregulated or downregulated by at least 1.5 folds after infection (**Figure 2A** and **Supplementary Data Sheet 1**). Our RT-qPCR results confirmed that the *Cxcl1* and *Cxcl2* mRNA levels were increased at 4 h post-infection but were decreased at 24 h post-infection (**Figure 2B**). In addition, *Il6, Il10*, and *Tnfα* mRNA levels were also increased at 4 h post-infection, then reduced at 24 h post-systemic *S. aureus* infection (**Figure 2C**).

To validate whether the transcriptionally upregulated cytokines/chemokines were produced by MZ B cells after bacterial infection, we performed *in vitro* culture experiments using MZ B cells stimulated for 24 h with *S. aureus* peptidoglycan (PGN), which is a major component of the bacterial cell wall that contributes to the maintenance of mechanical strength and cell integrity (45). Cytometric Bead Array analysis showed that the MZ B cells released a large amount of IL-6, together with low amounts of TNFα and IL-10 after PGN stimulation (**Figure 2D**). Next, to test the contribution of MZ B cells to the elevated IL-6 production following systemic *S. aureus* infection, we collected tissue fluid from WT and RBP-J CKO mouse spleens at various timepoints. Notably, the levels of IL-6 in WT mouse spleens were significantly higher than those in the spleens of RBP-J CKO mice at 3 and 6 h post-infection (**Figure 2E**). Consistent with this finding, we found a significantly elevated release of IL-6 by MZ B cells at 3 h after treatment with heat-killed *S. aureus* in culture (**Supplementary Figure 2A**). Moreover, intracellular IL-6 staining by flow cytometric analysis demonstrated that the frequency of IL-6-producing MZ B cells increased significantly after systemic *S. aureus* infection *in vivo* (**Supplementary Figures 2B, C**). Together, these results suggest that MZ B cells are a source of IL-6 in response to systemic *S. aureus* infection.

### Neutrophils Express Increased Levels of Multiple Chemokine Receptors After *S. aureus* Infection

**Figure 3:**
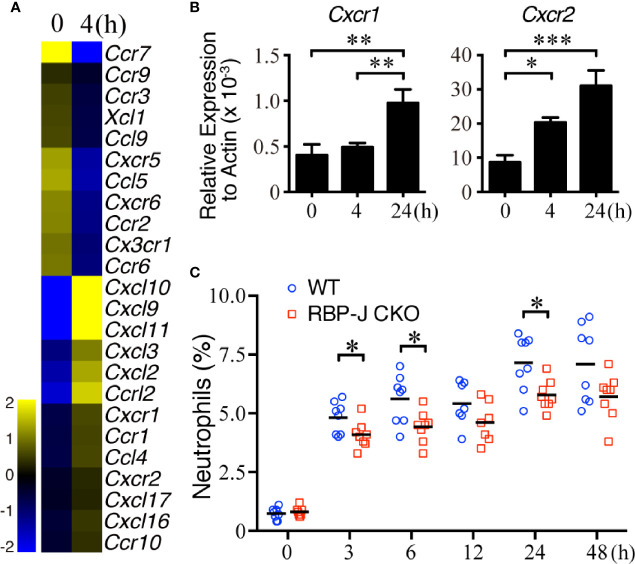
*Cxcr1/Cxcr2* levels in splenic neutrophils after systemic *S. aureus* infection. **(A)** Heatmap from a cDNA microarray analysis showing the changes in chemokine-related genes between splenic neutrophils isolated before and 4 h after infection with *S. aureus* (2.5 × 10^6^ CFU) by iv injection. The expression of chemokine-related genes with fold changes of >1.5 is shown. **(B)** RT-qPCR analysis showing the expression of *Cxcr1*/*Cxcr2* mRNA. Splenic neutrophils were isolated at 0, 4, or 24 h after *S. aureus* infection. Results were normalized to the actin mRNA levels. Three independent experiments were performed. Data are presented as the mean ± SEM (n = 6–8 mice per group). One-way ANOVA was used for comparison. **(C)** Flow cytometric analysis showing the frequency of splenic neutrophils (Ly6G^hi^CD11b^hi^) in WT and RBP-J CKO mice at various timepoints after *S. aureus* (2.5 × 10^6^ CFU) infection. Data are presented as the mean ± SEM (n = 7–8). An unpaired two-tailed *t*-test was performed. *p < 0.05, **p < 0.01, and ***p < 0.001.

Because IL-6 regulates systemic inflammatory responses partially by assisting with the infiltration of splenic leukocytes (46, 47), we next examined whether MZ B cells affect the recruitment of circulating neutrophils after systemic *S. aureus* infection. We first examined whether splenic neutrophils changed the expression of cytokine/chemokine genes after systemic *S. aureus* infection. Neutrophils isolated from untreated mice or mice infected with *S. aureus* 4 h previously were subjected to a cDNA microarray analysis. The results reveal that 24 chemokine genes were upregulated or downregulated by at least 1.5 folds in splenic neutrophils after infection (**Figure 3A** and **Supplementary Data Sheet 1**). CXCL1 and CXCL2 are members of the CXC chemokine family, and they bind with the CXC chemokine receptors CXCR1 and/or CXCR2 (48, 49). Our RT-qPCR results confirm that the *Cxcr1* and *Cxcr2* mRNA levels were significantly increased within 24 h after *S. aureus* infection (**Figure 3B**). Together, these data imply that the upregulated *Cxcr1*/*Cxcr2* in neutrophils may be a response to the enhanced expression of *Cxcl1*/*Cxcl2* in MZ B cells during the early recruitment phase after systemic *S. aureus* infection. In support of this possibility, we observed a significant increase in the percentage of splenic neutrophils soon after *S. aureus* infection in WT mice (**Figure 3C**). Notably, compared with *S. aureus*-infected WT mice, similarly infected RBP-J CKO mice had lower percentages of splenic neutrophils at 3, 6, and 24 h post-infection (**Figure 3C** and **Supplementary Figure 3**), suggesting that CXCL1/CXCL2 released by MZ B cells may recruit neutrophils in the early phase of systemic *S. aureus* infection.

### IL-6 and CXCL1/CXCL2 Are Important for Neutrophil Swarming to the MZ Area in Response to Systemic *S. aureus* Infection

**Figure 4:**
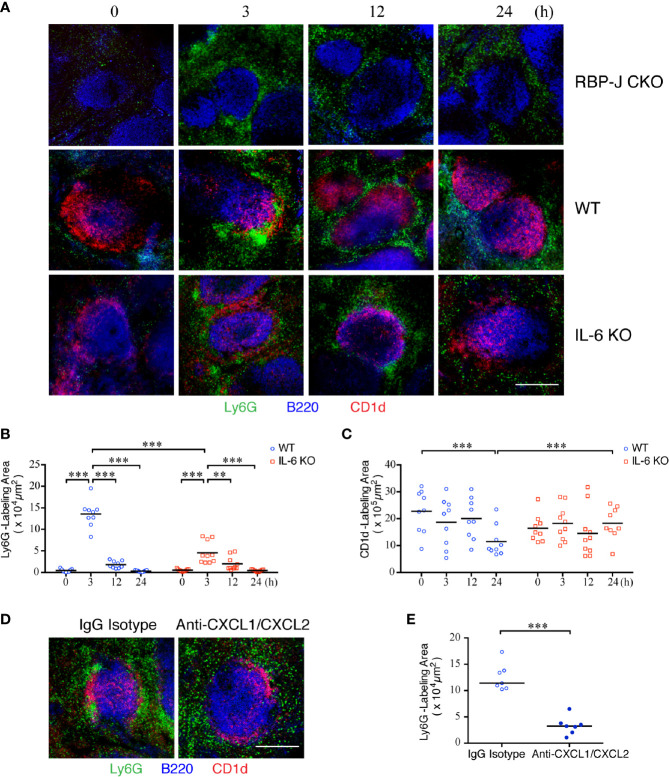
Kinetics of neutrophil swarming and regression in the MZ zone after *S. aureus* infection. **(A)** Confocal images of immunofluorescent staining of thick spleen sections showing the distribution of MZ B cells (red) and neutrophils (green) at 0, 3, 12, or 24 h after systemic *S. aureus* (2.5 × 10^6^ CFU) infection in RBP-J CKO, WT, or IL-6 KO mice. Scale bar = 200 µm. **(B, C)** MetaMorph software was used to calculate the area fluorescently labeled area by Ly6G per CD1d positive area **(B)** or CD1d **(C)** inside the white pulp of WT or IL-6 KO mice from the confocal microscopy images in **(A)**. **(D)** Confocal images of immunofluorescence staining of spleen sections at 3 h after systemic *S. aureus* (2.5×10^6^ CFU) infection showing the numbers of neutrophils (green) in WT mice intravenously injected with anti-CXCL1/CXCL2 or IgG control antibodies one h prior to infection. Scale bar = 200 µm. **(E)** MetaMorph software was used to calculate the Ly6G fluorescently labeled area of each CD1d-positive area in the white pulp of WT mice from the confocal microscopy images in **(D)**. Results were analyzed by performing an unpaired *t*-test. Data are presented as the mean ± SEM (n = 9–10). **p < 0.01, and ***p < 0.001.

We next sought to examine the influence of cytokines and chemokines, on the dynamics of neutrophil infiltration into the spleen after *S. aureus* infection. We first employed confocal imaging using thick sections to define the location of the MZ B-cell area, distinguished by CD1d^+^ in staining; it was located at the junction of the red pulp and white pulp of the spleen, separated from the follicle area (**Supplementary Figure 4A**). The number of neutrophils, as defined by Ly6G^+^ staining, in the red pulp was small in the uninfected mice (**Figure 4A**). Notably, we observed that neutrophils swarmed and packed closely together with MZ B cells in the spleens of WT mice at 3 h post-infection. This enhanced amount of neutrophil accumulation was reduced in RBP-J CKO mice, indicating that MZ B cells are important for the early recruitment of neutrophils during systemic *S. aureus* infection (**Figure 4A**).

Because the number of neutrophils at the outer ring of the MZ declined at 12 and 24 h after systemic *S. aureus* infection (**Figure 4A**), when we observed that the IL-6 levels also reduced after *S. aureus* infection (**Figure 2E**), we speculated that the formation of a large number of neutrophil clusters may occur in response to the elevated expression of IL-6 in MZ B cells. In support of this idea, the number of neutrophils in *Il-6* knockout (IL-6 KO) mice at 3 h post-infection appears to be lower than that in WT mice (**Figure 4A**). A quantitative analysis performed by using MetaMorph software further revealed that the total area of neutrophil accumulation near the MZ area was significantly higher at 3 h post-infection in WT mice as compared with those at later timepoints (**Figure 4B** and **Supplementary Figure 4B**). Notably, the total area of neutrophil clustering near the MZ area was smaller in IL-6 KO mice compared with that in WT mice (**Figure 4B** and **Supplementary Figure 4C**), indicating that IL-6 promotes the initial neutrophil swarming to MZ areas during systemic *S. aureus* infection. Furthermore, the CD1d-labeled area in the spleens of WT mice was significantly smaller at 24 h post-infection as compared with that in uninfected mice (**Figure 4C**). However, in IL-6 KO mice, the size of the CD1d-labeled area was not significantly different between uninfected and *S. aureus*-infected mice, suggesting that IL-6 may affect the activation and differentiation of MZ B cells. To test whether chemotactic signals also contribute to neutrophil recruitment during systemic *S. aureus* infection, we injected anti-CXCL1 and anti-CXCL2 antibodies to neutralize the effect of CXCL1/CXCL2 one h before systemic *S. aureus* infection. Confocal imaging with thick tissue sections showed that, following depletion of the activity of CXCL1/CXCL2, neutrophils were distributed in the red pulp and much less neutrophils were present in the MZ area 3 h after *S. aureus* infection, as compared with those in infected mice injected with isotype control antibody (**Figure 4D**). A quantitative analysis performed by using MetaMorph software indicated that depletion of CXCL1/CXCL2 before infection prevents neutrophil recruitment to the MZ (**Figure 4E** and **Supplementary Figure 4D**). Together, these results indicate that IL-6 and CXCL1/CXCL2 contribute to neutrophil swarming during the early phase of systemic *S. aureus* infection.

### The Neutrophil–MZ B Cell Interaction Promotes IgM Production

**Figure 5:**
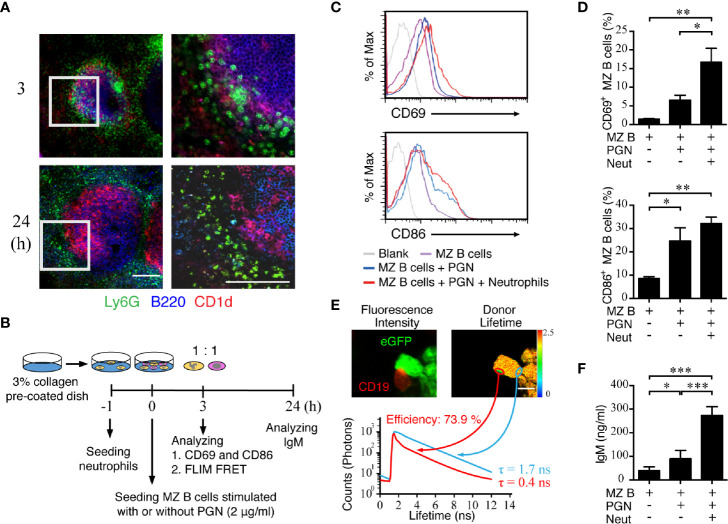
Activation of MZ B cells and IgM production after interaction with neutrophils in *S. aureus* infection. **(A)** Confocal microscopy images showing the neutrophils and MZ B cells at 3 h or 24 h after systemic infection with *S. aureus* (2.5 × 10^6^ CFU) in WT mice. Right panels present enlarged images from the boxed areas in the left panels. Scale bar = 100 µm. **(B)** Schematic diagram showing the experimental design of MZ B cell and neutrophil co-culture experiments. **(C)** Flow cytometric analysis showing the levels of CD69 (upper panel) and CD86 (lower panel) expression on MZ B cells co-cultured with neutrophils at 3 h after stimulation with PGN (2 µg/ml). **(D)** Statistical analysis of the percentages of CD69^+^ (upper panel) or CD86^+^ (lower panel) MZ B cells co-cultured with neutrophils (Neut) with or without PGN stimulation. **(E)** The interaction of neutrophils and MZ B cells detected by FLIM FRET analysis. Neutrophils were purified from LysM-eGFP mice. Sorted MZ B cells were labeled with anti-CD19 primary antibody and Alexa Fluor 546 secondary antibody. The confocal fluorescence intensity (left panel) and average donor lifetime (right panel) of neutrophils were measured. Lifetime values were pseudocolored according to the color scale. Histograms show the lifetime values of the junction area of neutrophils and MZ B cells (red histogram) as compared with neutrophils only (blue histogram), in a representative experiment. Scale bar = 5 µm. **(F)** ELISA showing the levels of IgM in supernatants of MZ B cells co-cultured with neutrophils for 24 h with or without PGN stimulation. Results were analyzed by an unpaired *t*-test. Data are analyzed by one-way ANOVA and presented as the mean ± SEM (n = 4 in D, and n = 6 in F). *p < 0.05, **p < 0.01 and ***p < 0.001.

Because our results from the neutrophil staining in thick spleen tissue sections demonstrated a suppressed neutrophil accumulation in the MZ area of RBP-J CKO mice, we next examined the molecular consequence of an interaction between neutrophils and MZ B cells. The results from CD1d and Ly6G staining show that neutrophil clusters were in close contact with MZ B cells at 3 h after systemic *S. aureus* infection, whereas neutrophils were scattered around the outer ring of MZ B cells at 24 h post-infection (**Figure 5A**). Furthermore, confocal images of the locations of neutrophils and MZ B cells in a thick tissue section of WT spleen from a mouse infected with *S. aureus* 3* h* previously showed a colocalization rate of 47% (**Supplementary Figures 5A** and **5B**). To further understand the consequence of the interaction between neutrophils and MZ B cells, we conducted co-culture experiments and examined whether an interaction between neutrophils and MZ B cells promotes antibacterial responses (**Figure 5B**). First, we performed a flow cytometry analysis to measure the expression levels of the costimulatory molecule CD86 and the early activation molecule CD69 on MZ B cells after they had been co-cultured with neutrophils for 3 h in the presence or absence of PGN stimulation (**Figure 5C**). We found that the CD86 and CD69 expressions on MZ B cells were elevated after PGN stimulation and that CD69 elevation was further promoted in the presence of neutrophils (**Figure 5D**). However, treatment with heat-killed *S. aureus* significantly elevated the expression of CD69 and CD86 on MZ B cells, even in the absence of neutrophils (**Supplementary Figures 6A–C**). We next assessed the interaction between neutrophils and MZ B cells by using a FLIM-FRET analysis. Confocal imaging indicated that an enrichment of CD19-labeled signal clustered on the surface of MZ B cells at the neutrophil junction following PGN stimulation (**Figure 5E**, red circle). We used the average photon arrival time to represent the mean lifetime. Our results reveal that neutrophils (donor) in contact with an MZ B cell (acceptor) indeed have a shorter fluorescent lifetime compared with neutrophils not in contact with an MZ B cell. A quantitative analysis of the FRET efficiency estimates it at 73.9% (**Figure 5E**). These results suggest that there is a direct interaction between neutrophils and MZ B cells. The amount of IgM produced by activated MZ B cells after their co-culture with neutrophils was also measured by ELISA. We found that MZ B cells co-cultured with neutrophils rapidly (within a day) released significantly higher amounts of IgM after stimulation with PGN compared with similarly treated MZ B cells cultured alone (**Figure 5F**). Similarly, co-culture of neutrophils with MZ B cells significantly enhances the production of IgM after the treatment with heat-killed *S. aureus* (**Supplementary Figure 6D**). Together, these combined results provide evidence for the direct interaction of neutrophils and MZ B cells and for the enhanced differentiation of IgM-producing cells by neutrophils after *S. aureus* infection.

### Neutrophil Depletion Affects MZ B-Cell Differentiation After Bacterial Infection

**Figure 6:**
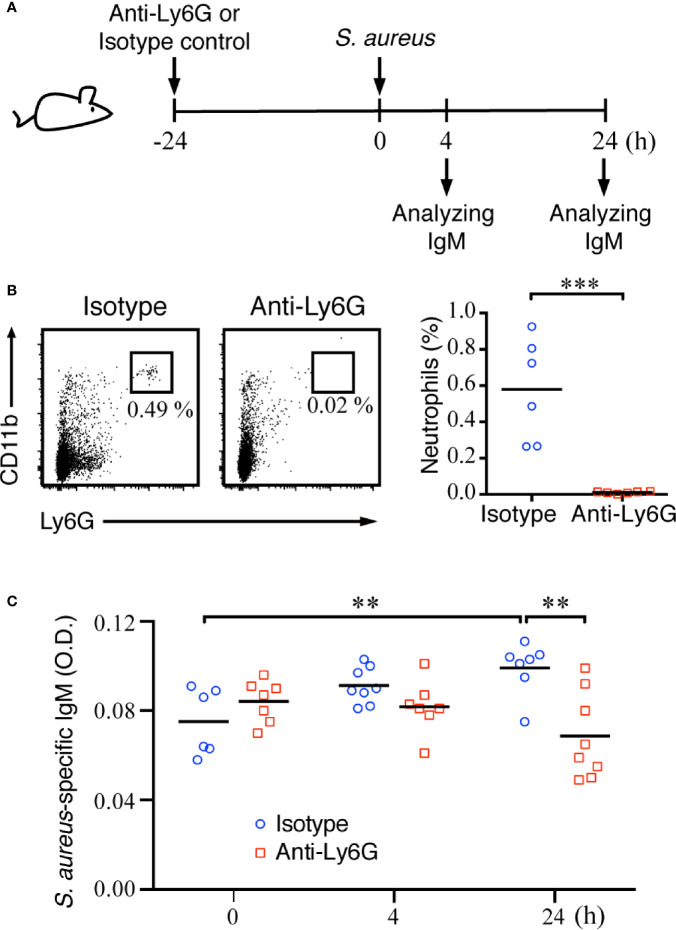
Effect of neutrophil deficiency on the activation of MZ B cells and IgM production in *S. aureus*-infected mice. **(A)** Schematic diagram of the experimental design. **(B)** Flow cytometry analysis showing the depletion of neutrophils (Ly6G^hi^CD11b^hi^) in the spleens of C57BL/6 mice at 24 h after an intraperitoneal injection of anti-Ly6G antibody. **(C)** ELISA analysis showing the levels of *S. aureus*-specific IgM in the splenic tissue fluids of C57BL/6 mice injected with isotype control antibody or anti-Ly6G antibody. Results were analyzed by an unpaired Student’s *t*-test. Data are presented as the mean ± SEM (n = 6 in B, and n = 6–8 in C). **p < 0.01 and ***p < 0.001.

To confirm whether neutrophils have important roles in assisting with MZ B-cell activity during the early phase of systemic *S. aureus* infection, we next depleted neutrophils and assessed whether the MZ B-cell differentiation was affected after *S. aureus* infection (**Figure 6A**). Mice injected with anti-Ly6G antibody showed a robust reduction of neutrophils as compared with mice injected with the isotype control antibody (**Figure 6B**). ELISAs were performed on splenic tissue fluids to analyze the amount of *S. aureus*-specific IgM at 4 and 24 h post-infection. Our results show a slightly induced production of *S. aureus*-specific IgM in control mice at 24 h post-infection, whereas mice depleted of neutrophils had significantly lower levels of *S. aureus*-specific IgM at 24 h post-infection (**Figure 6C**). Thus, neutrophils play an important role in promoting the activation and differentiation of MZ B cells during acute *S. aureus* infection.

## Discussion

Previous studies have noted that MZ B cells not only participate in innate immune responses but also mediate T cell-dependent immunity (50). Our findings here demonstrate the role of MZ B cells in the regulation of innate immunity during systemic bacterial infection, which involves in their interaction with neutrophils. We show here that, compared with WT mice, MZ B cell-deficient mice exhibited exacerbated systemic bacterial infections with reduced survival rates, which was linked with their reduced neutrophil accumulation during the early phase of systemic *S. aureus* infection.

We here use RBP-J CKO mice to study the role of MZ B cells in systemic *S. aureus* infection. Although activation of Notch pathway has been shown to promote B cell differentiation (51), RBP-J CKO mice did not show obvious changes in Ig production in response to model antigen immunization, as compared with those produced in control mice (3). Therefore, the reduced production of IgM after systemic *S. aureus* infection in RBP-J CKO mice may not result from the effects of altering Notch signaling during plasma cell differentiation. Also, the reduced IgM production after systemic *S. aureus* infection in RBP-J CKO mice may not attribute to the changes in the population of macrophages because RBP-J CKO mice still showed the normal presence of metallophilic macrophages adjacent to the MZ in the spleen (3, 52). We found that IL-6 is important for neutrophil swarming during systemic *S. aureus* infection and that MZ B cells may be an important cellular source of IL-6 in this context. The roles of IL-6 in B-cell immunity have been extensively studied. For example, in combination with a proliferation-inducing ligand (APRIL) or stem cell-soluble factor, IL-6 can promote the production of immunoglobulin by long-lived plasma cells in the bone marrow (53, 54). IL-6 produced by B cells also contributes to the formation of spontaneous germinal centers in autoimmunity (55). Here, we observed that IL-6 levels in the spleen were largely reduced in RBP-J CKO mice, compared with WT mice, during the early phase of systemic *S. aureus* infection. We suspect that this robust reduction in IL-6 production might at least partly result from the lack of MZ B cells. However, it has been shown that IL-6-driven signaling restrains the recruitment of neutrophils in an animal model of acute peritoneal inflammation. STAT3 activation mediated by gp130 reduces the expression of neutrophil-activating chemokines CXCL1/KC and affects neutrophil clearance (56). IL-6 produced by MZ B cells accounts for the proinflammatory role of MZ B cells during endotoxic shock through IgM Fc receptor (FcμR)-coupled TLR4 signaling (13). In the present study, PGN from *S. aureus* may initiate TLR2-mediated signaling to induce IL-6 production by MZ B cells. We show that IL-6 is beneficial for the hosts during systemic *S. aureus* infection because IL-6 has a positive role in neutrophil swarming during the acute phase. It is still poorly understood how IL-6 signaling can act as a double-edged sword to regulate the innate immune responses under different conditions. Here, we speculate that following *S. aureus* infection, activated MZ B cells may release the chemokine CXCL2 to attract many neutrophils that have upregulated expression of their CXCR2 receptor, *Cxcr1*/*Cxcr2*. In support of this possibility, we found a correlation between lower numbers of neutrophils surrounding the MZ area with lower levels of CXCL2 expression by MZ B cells at 24 h after systemic *S. aureus* infection. We further demonstrated that CXCL1/CXCL2 released by MZ B cells also participated in the recruitment of neutrophils after systemic *S. aureus* infection. Prior studies have noted that CXCL1/CXCL2 are the main neutrophil-attracting chemokines in group B Streptococcus (GBS)-induced neutrophil recruitment. Specifically, neutrophils can employ a positive feedback mechanism driven by high levels of CXCL2 to enhance their recruitment to the sites of infection and antibacterial activity (57). We suspect that the increased CXCL1/CXCL2 expression by neutrophils after *S. aureu*s infection may also act in an autocrine manner to attract more neutrophils and enhance antibacterial activities.

Neutrophils are a heterogeneous and plastic innate cell population. Inflammatory cytokines, including GM-CSF, or co-culture with antigen and T cells can induce the expression of MHCII on neutrophils, thereby triggering the antigen-presenting functions of neutrophils (58, 59). However, the underlying molecular mechanisms for antigen presentation crosstalk between neutrophils and MZ B cells remain unclear. Previous studies using FRET analysis reported that antibody-induced signals from Transmembrane Activator and CAML Interactor (TACI) and TLR synergistically activate MZ B cells and induce plasmablast differentiation through a rapamycin-sensitive pathway (60). For a long time, it has been reported that MZ B cells integrate signals from B-cell receptors (BCR), complement receptors (C3), and Toll-like receptors to rapidly activate antigen-specific IgM (8, 61). In the co-culture experiment, compared with PGN stimulated MZ B cells, we found that treatment with heat-killed *S. aureus* was able to effectively increase the expression of CD69 and CD86 on MZ B cells. We speculate that, in addition to activating TLR2 on MZ B cells through PGN, *S. aureus* contains other molecules to be recognized by MZ B cells. However, differentiation of MZ B cells into Ig producing cells needs the help from neutrophils. Here, we performed a FLIM-FRET analysis by using confocal microscopy, the results of which demonstrate an interaction between MZ B cells and neutrophils at 3 h after stimulation with the cell wall component of *S. aureus.* We found that the direct cell-cell communication between activated neutrophils and MZ B cells was related to CD19 expression. However, the exact molecules involved in this direct interaction between MZ B cells and neutrophils requires further study. Nevertheless, our data, along with a previous report showing that IL-6-induced STAT3 activation enhances the recruitment of neutrophils and contributes to host defense against *E. coli*-induced pneumonia, support the idea that neutrophils possess multiple functions to assist in the clearance of bacterial infection in the spleen (62). We found that mice depleted of neutrophils were more susceptible to systemic *S. aureus* infection as compared with mice treated with isotype control antibodies. The spleens of the neutrophil-depleted mice were nearly devoid of infiltrating neutrophils, and 27.5% of these mice died within 24 h of infection with *S. aureus* (**Supplementary Figure 7**). Thus, our findings are similar to the results from a previous study demonstrating that systemic TLR2 activation and bone marrow granulocyte depletion in mice exacerbated *Listeria monocytogenes* infection and led to uncontrolled bacterial propagation (63).

The production of high affinity antibodies following infection-induced germinal center reactions usually requires several days to emerge. MZ B cells fill the gap before the peak germinal center reactions by deploying innate immune defenses and rapidly producing antibodies. The present work demonstrates the requirement for IL-6 and CXCL1/CXCL2 to attract an accumulation of neutrophils. This direct interaction between MZ B cells and neutrophils in turn assists with the maturation of MZ B cells as they differentiate into antibody-secreting cells during the early phase of bacterial infection. Thus, MZ B cells possess the regulatory functions necessary to orchestrate neutrophil swarming during the early stage of systemic bacterial infection. Our results may provide some clues on how better to control systemic *S. aureus* infection.

## Data Availability Statement

The datasets presented in this study can be found in online repositories. The names of the repository/repositories and accession number(s) can be found in the article/**Supplementary Material**.

## Ethics Statement

The animal study was reviewed and approved by Institutional Animal Care and Use Committee (IACUC) of Academia Sinica.

## Author Contributions

K-IL conceived and designed the study. L-WL, C-WC, M-FC, and I-YL performed the experiments and analyzed the data. L-WL and K-IL wrote the manuscript. All authors contributed to the article and approved the submitted version.

## Funding

This work was supported by grants from Academia Sinica (AS-105-TP-B-08-01,AS-IA-107-L05), Taiwan, and Ministry of Science and Technology (MOST 109-2320-B-001-023-MY3).

## Conflict of Interest

The authors declare that the research was conducted in the absence of any commercial or financial relationships that could be construed as a potential conflict of interest.
