# Supplementary material for: Marginal Zone B Cells Assist With Neutrophil Accumulation to Fight Against Systemic Staphylococcus aureus Infection
Source: Front Immunol. 2021 May 10;12:636818. doi: 10.3389/fimmu.2021.636818 (PMC8141640; doi:10.3389/fimmu.2021.636818)

Marginal zone B cells assist with neutrophil accumulation to fight against systemic *Staphylococcus aureus* infection

Supplementary Material

Supplementary Figure Legends

Supplementary Figure 1. MZ B cells differentiate into plasma cells after systemic *S. aureus* infection. (**A**) Dot plots from a flow cytometry analysis showing the percentage of splenic $CD138^+$ plasma cells from WT and RBP-J CKO mice on various hours after they were intravenously inoculated with 2.5×10^6 CFU of *S. aureus.* (**B**) Dot plots from a flow cytometry analysis showing the percentage of splenic $CD138^+Ly6C^+$ plasma cells from WT and RBP-J CKO mice 24 h after *S. aureus* (2.5×10^6 CFU) infection. Data are representative of at least three independent experiments.

Supplementary Figure 2. MZ B cells produce IL-6 in response to *S. aureus*. (**A**) MZ B cells were sorted from spleens of WT mice by cell sorter. Sorted MZ B cells were cultured with or without heat-killed *S. aureus* (MOI=10) for 3 h, followed by measuring the levels of IL-6 in culture supernatants by ELISA. (**B**) WT mice intravenously infected with 2.5×10^6 CFU of *S. aureus*. Dot plots from a flow cytometry analysis showing the percentage of IL-6-producing MZ B ($B220^+CD21/35^{hi}CD23^{lo}$) cells in WT mice at 24 h after *S. aureus* infection. (**C**) Statistical analysis of the percentages of IL-6-producing MZ B cells in (B). The statistical analysis was conducted by performing an unpaired *t*-test (n = 3–5). Data are presented as the mean ± SEM. $^{**}p < 0.01$.

Supplementary Figure 3. Kinetics of the splenic neutrophil percentage after systemic *S. aureus* infection. Dot plots from a flow cytometry analysis showing the percentage of splenic neutrophils ($Ly6G^{hi}CD11b^{hi}$) from WT and RBP-J CKO mice at various timepoints after they were intravenously inoculated with 2.5×10^6 CFU of *S. aureus.* Data are representative of at least three independent experiments.

Supplementary Figure 4. Confocal imaging analysis identifying the Ly6G-labeled area and CD1d-labeled area in the MZ at the indicated timepoints after systemic *S. aureus* infection. (**A**) Overview of the confocal microscopy imaging of spleen thick sections isolated after systemic *S. aureus* infection. Splenic thick sections from WT mice infected with 2.5×10^6 CFU of *S. aureus* for 24 h were subjected to staining with antibodies against B220 (blue), Ly6G (green), and CD1d (red), followed by confocal imaging analysis. White pulp and red pulp are marked with yellow and orange arrows, respectively. MZ B cells are localized at the junction of the white and red pulps and are characterized by CD1d expression (red). Scale bar = 200 µm. (**B, C**) Spleen sections from WT mice (B) or IL-6 KO mice (C) extracted at 0, 3, 12, or 24 h after infection with 2.5×10^6 CFU of *S. aureus* were stained with antibodies against B220 (blue), Ly6G (green), and CD1d (red). **(D)** Spleen sections of WT mice extracted at 3 h after *S. aureus* ($2 .5 \times 10^6$ CFU) infection were stained with antibodies against B220 (blue), Ly6G (green), and CD1d (red). Mice were pretreated by intravenous injection of anti-CXCL1/CXCL2 antibodies or IgG isotype control antibody 1 h prior to infection. The white pulps in the confocal microscopy images of the spleen are indicated by a yellow circle. CD1d-labeled areas and Ly6G-labeled areas in the white pulp were quantified using MetaMorph software. Scale bar = 200 µm.

Supplementary Figure 5. Confocal images of MZ B cell and neutrophil localization. (**A**) Confocal images of splenic thick sections from WT mice that had been intravenously inoculated with 2.5×10^6 CFU of *S. aureus* 3 h previously. The tissue sections were subjected to staining with antibodies against Ly6G (green) and CD1d (red). (**B**) A scatter plot showing the corresponding fluorescence colocalization area of MZ B cells and neutrophils, as indicated by a white gate. Scale bar = 200 µm.

Supplementary Figure 6. Activation of MZ B cells and IgM production after interaction with neutrophils and heat-killed *S. aureus*. (A) Schematic diagram showing the experimental design of MZ B cell and neutrophil co-culture experiments with heat-killed *S. aureus* (MOI=10). **(B)** Flow cytometry analysis showing the levels of CD69 (upper panel) and CD86 (lower panel) expression on MZ B cells co-cultured with neutrophils at 3 h after treatment with heat-killed *S. aureus*. **(C)** Statistical analysis of the percentages of $CD69^+$ (upper panel) or $CD86^+$ (lower panel) MZ B cells co-cultured with neutrophils (Neut) with or without heat-killed *S. aureus* treatment. **(D)** ELISA showing the levels of IgM in supernatants of MZ B cells co-cultured with neutrophils together with or without heat-killed *S. aureus* (MOI=10) stimulation for 24 h. Results were analyzed by an unpaired *t*-test. Data are analyzed by one-way ANOVA and presented as the mean ± SEM (n = 3 in C, and n =3~4 in D). $*p < 0.05$, $**p < 0.01$ and $***p < 0.001$.

Supplementary Figure 7. Effect of neutrophil depletion on the survival of *S. aureus*-infected mice. The survival rates of mice injected with anti-Ly6G or isotype control antibodies were recorded at 4 and 24 h after they had been intravenously injected with *S. aureus.* An injection of anti-Ly6G antibody (400 µg per mouse) was used to deplete the neutrophils in the spleens of WT mice at 24 h before the animals were infected with 2.5×10^6 CFU of *S. aureus.* Data are mean ± SEM from three independent experiments. Each experiment contained at least four mice per group. Results were analyzed by an unpaired *t*-test. Data are presented as the mean ± SEM. $**p < 0.01$.

Lo et al. Supp Fig. 1

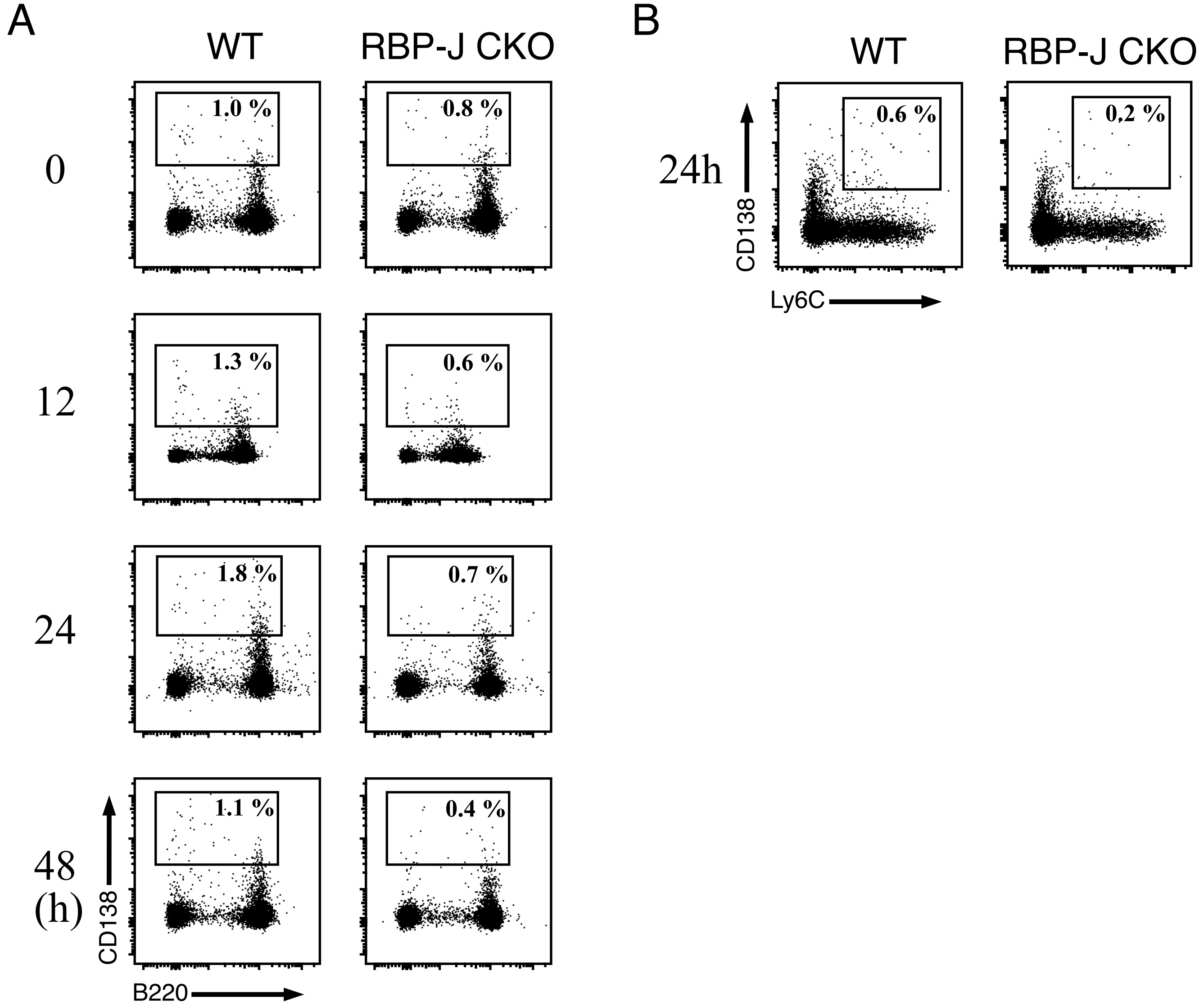

Lo et al. Supp Fig. 2

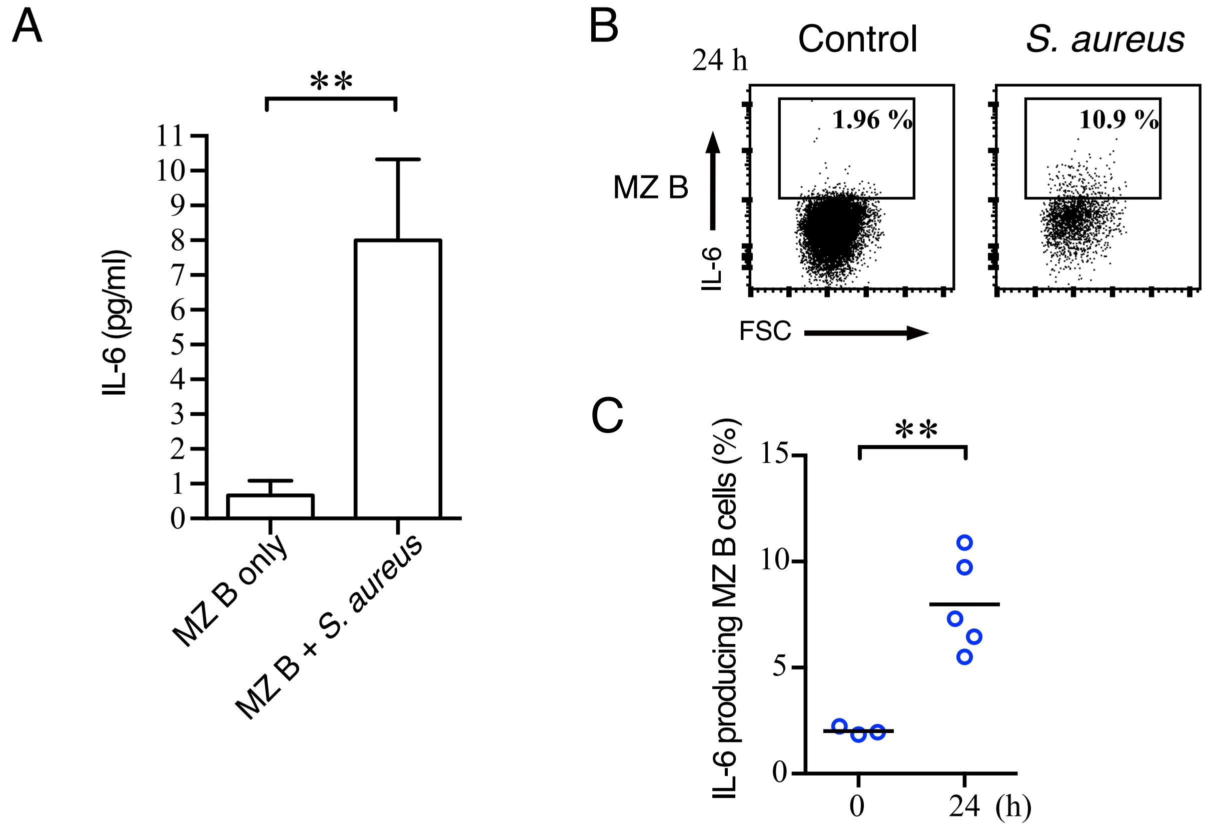

Lo et al. Supp Fig. 3

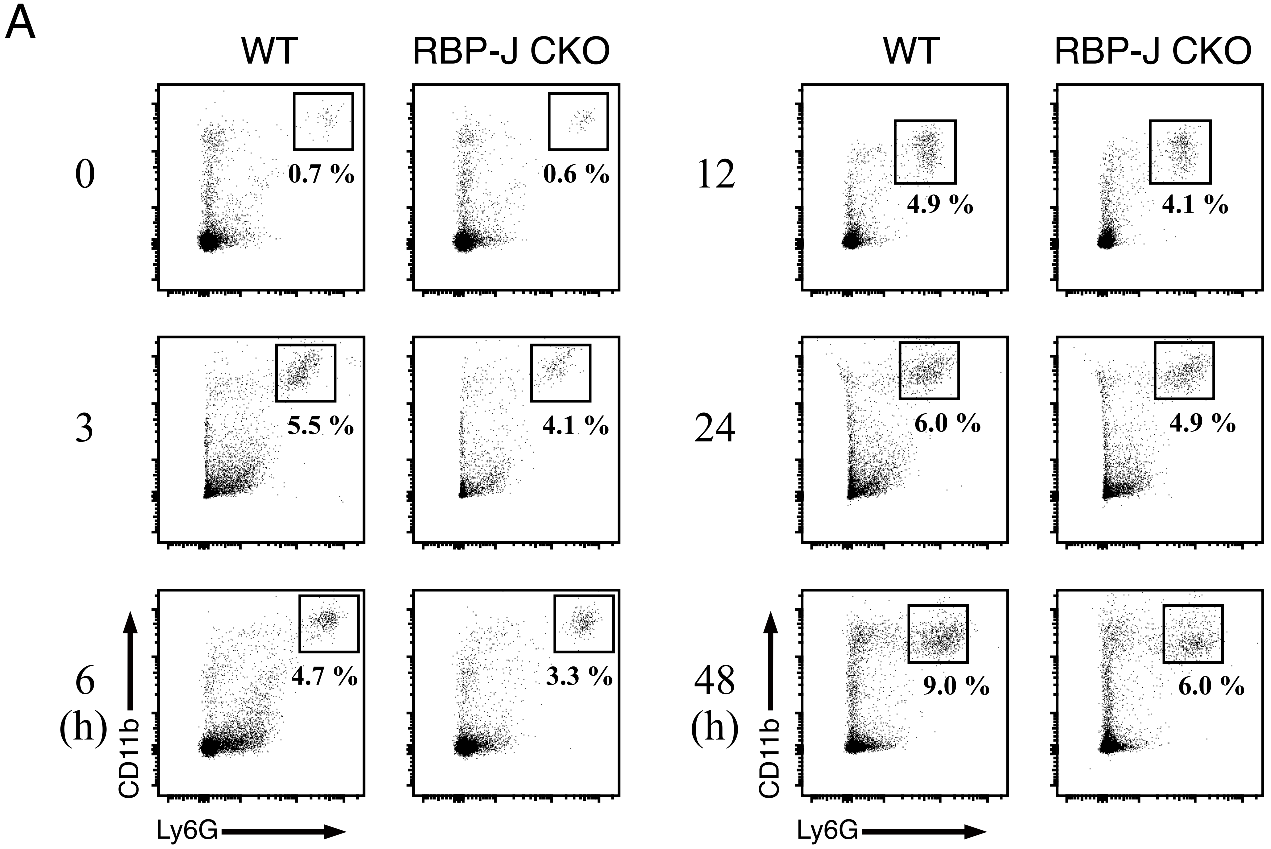

Lo et al. Supp Fig. 4

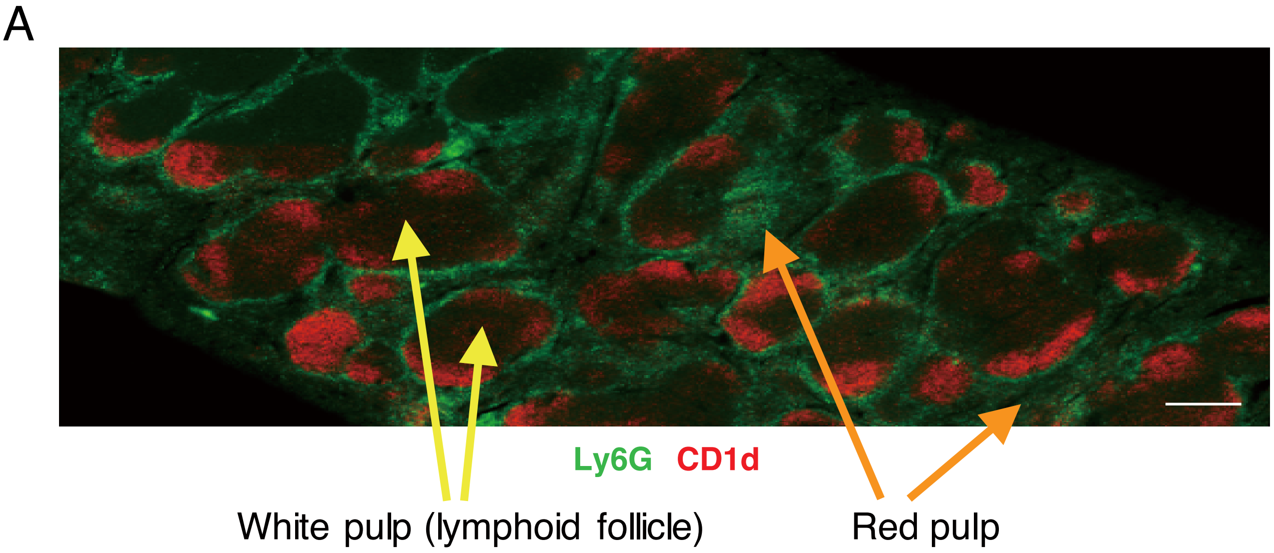

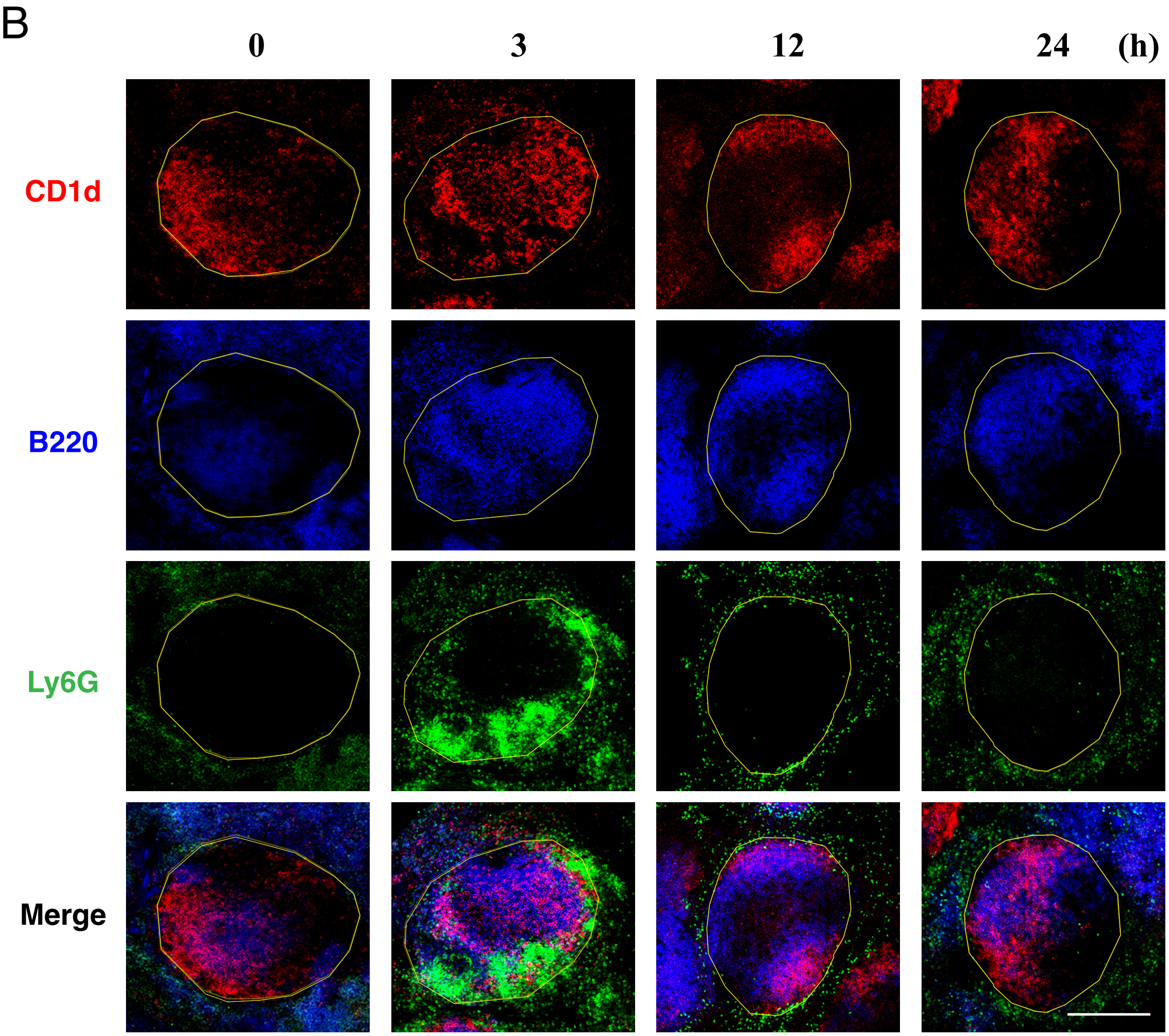

Lo et al. Supp Fig. 4

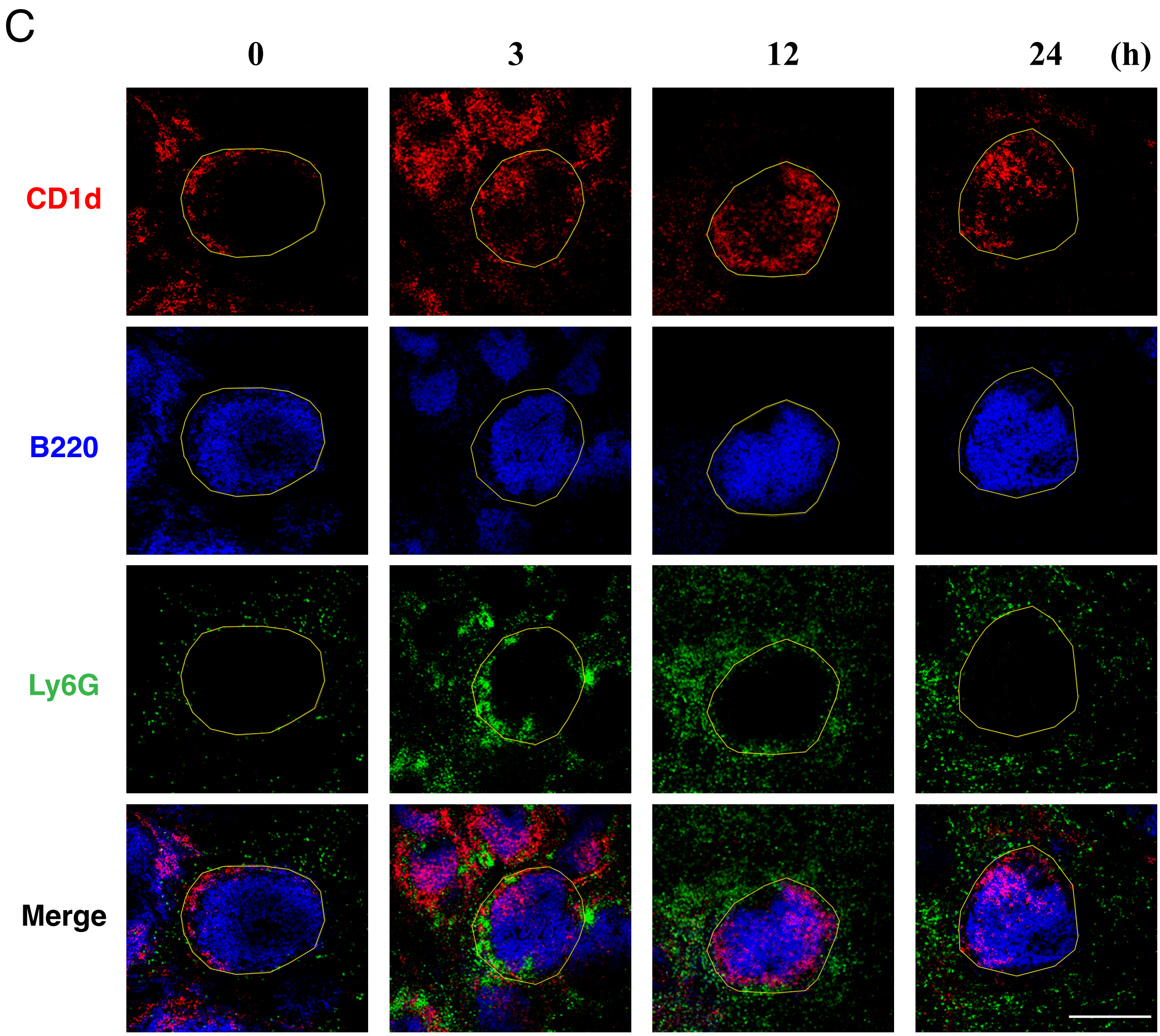

Lo et al. Supp Fig. 4

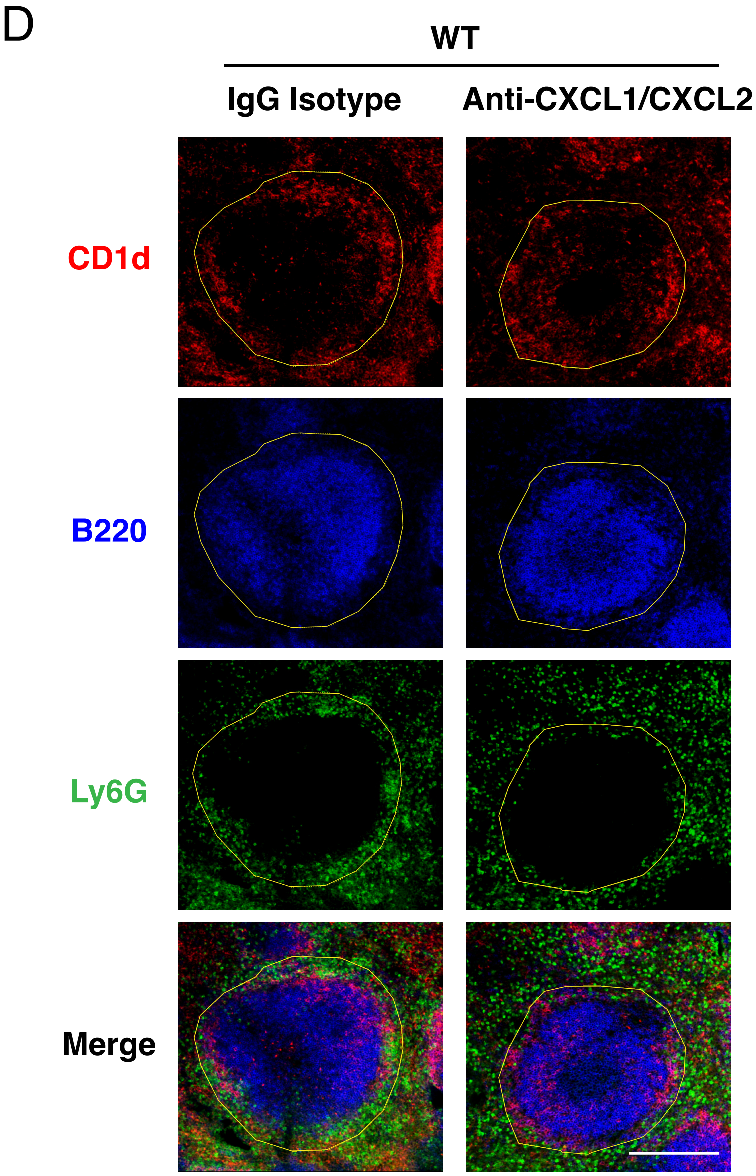

Lo et al. Supp Fig. 5

A

Ly6G

CD1d

Merge

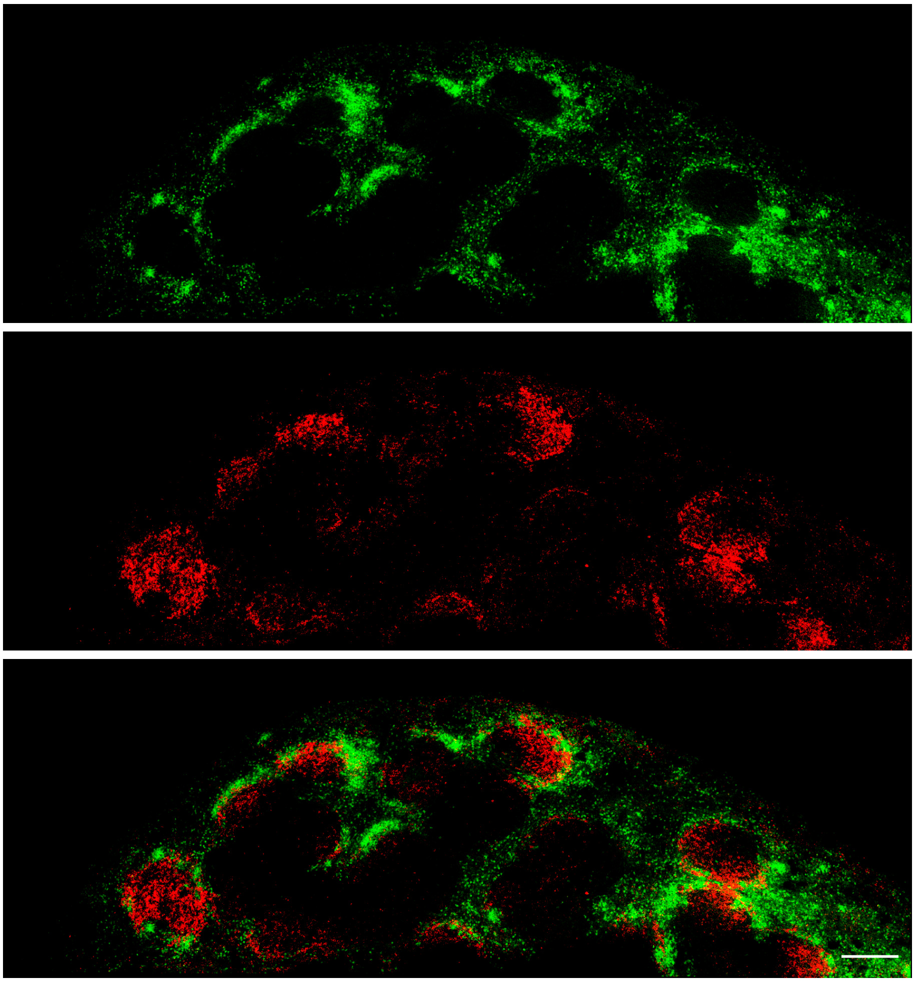

B

Co-locolization

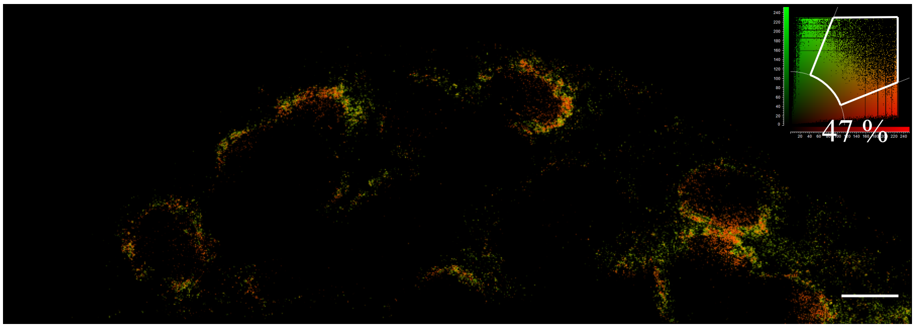

Lo et al. Supp Fig. 6

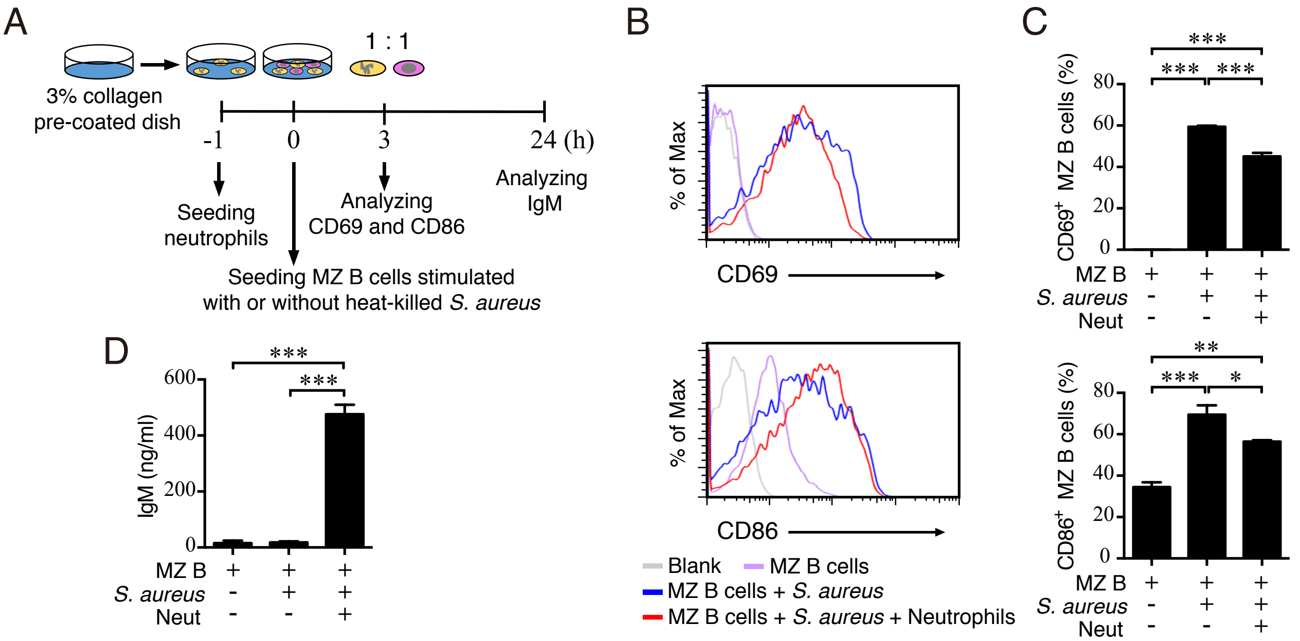

Lo et al. Supp Fig. 7

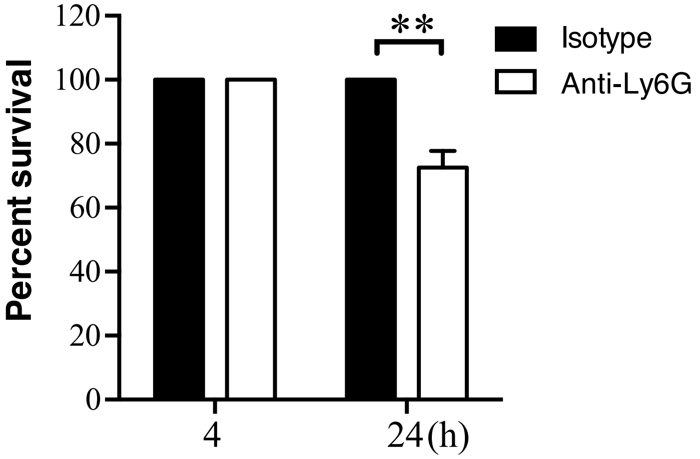

Supplement: Supplementary file 2 [file DataSheet_2.pdf]
